# miR-21 regulates ischemic neuronal injury via the p53/Bcl-2/Bax signaling pathway

**DOI:** 10.18632/aging.203530

**Published:** 2021-09-22

**Authors:** Honglin Yan, Wenxian Huang, Jie Rao, Jingping Yuan

**Affiliations:** 1 Department of Pathology, Renmin Hospital of Wuhan University, Wuhan 430060, Hubei, P.R. China

**Keywords:** cerebral ischemia, neuronal injury, miR-21, p53, Bcl-2/Bax

## Abstract

Focal cerebral ischemia leads to a large number of neuronal apoptosis, and secondary neuronal death is the main cause of cerebral infarction. MicroRNA-21 (miR-21) has been shown to be a strong anti-apoptosis and pro-survival factor in ischemia. However, the precise mechanism of miR-21 in ischemic neuroprotection remains largely unknown. In this study, miR-21 was down-regulated while p53 was up-regulated following ischemia *in vitro* and *in vivo*. Overexpression of miR-21 *in vitro* and *in vivo* substantially inhibited the expression of p53 following ischemia, while inhibition of miR-21 *in vitro* and *in vivo* promoted p53 expression following ischemia. Moreover, the miR-21/p53 axis regulated the expression of Bcl-2/Bax and abolished OGD/R-induced neuronal injury *in vitro*. Furthermore, overexpression of miR-21 *in vivo* reduced neuronal death, protected against ischemic damage, and improved neurological functions by inhibiting p53/Bcl-2/Bax signaling, while inhibition of miR-21 enhanced the p53/Bcl-2/Bax signaling and aggravated the ischemic neuronal injury *in vivo*. Our data uncover a novel mechanism of miR-21 in regulating cerebral ischemic neuronal injury by inhibiting p53/Bcl-2/Bax signaling pathway, which suggests that miR-21/p53 may be attractive therapeutic molecules for treatment of ischemic stroke.

## INTRODUCTION

During cerebral ischemia, insufficient blood supply to the brain and ischemia-reperfusion will irreversibly damage brain tissue and neurons [1, 2]. Focal cerebral ischemia induces a large number of neuronal apoptosis in the ischemic core, and secondary neuronal death plays a leading role in the long-term neurological damage after cerebral infarction [3, 4]. Up to now, the underlying molecular mechanism that determines ischemic neuronal death is still not entirely understood. MicroRNA (miRNA) is a class of non-coding RNA that promotes the degradation of mRNA or inhibits the translation of mRNA via complementary binding with the 3’-UTR of its target mRNA, thus inhibiting the regulation of target protein [5]. Recent studies have demonstrated that multiple miRNAs altered during cerebral ischemia *in vitro* and in *vivo,* suggesting that miRNAs may be key regulators in the progression of ischemic stroke [2, 6–8].

Among the miRNAs identified following ischemic stroke, miRNA-21 (miR-21) has been demonstrated to be a powerful factor that inhibits apoptosis and promotes survival [7–9]. A study by Zhou et al. showed that miR-21 mainly played a role in inhibiting cell apoptosis following oxygen-glucose deprivation and reoxygenation (OGD/R) in N2a neuroblastoma cells [8]. Buller et al. revealed that overexpression of miR-21 in embryonic neurons cultured *in vitro* inhibited OGD/R-induced apoptosis [9]. Our previous study has reported that miR-21 was decreased in the foci of the focal cerebral infarction in mice and in N2a cells exposed to OGD [10]. These findings suggest that miR-21 provides neuroprotection during ischemic brain injury and may be a feasible target for the treatment of ischemic stroke. However, the precise mechanism of miR-21 in ischemic neuroprotection is not completely understood.

The p53 protein is a transcription factor controlling transcriptional-dependent apoptosis and necrosis. Studies have shown that cerebral ischemic neuronal injury was closely related to the activation of p53 [10, 11]. Application of p53 inhibitors or p53 deficiency significantly reduced the damage in various stroke models [10, 11]. It seems that p53 plays an important role in cerebral ischemic injury and has become a therapeutic target against stroke [11]. Accumulate evidence showed that p53-mediated neuronal apoptosis occurred through a variety of molecular mechanisms, in which the activation of p53 in ischemic injury was regulated by various non-coding RNAs (ncRNAs) [11–14]. It is worth noting that several miRNAs are involved in the p53 pathway. They directly inhibit p53 or its downstream target proteins (such as Bax, Bcl-2, Fas, and FasL), indicating the key role of miRNAs in the p53 pathway [15–17]. However, the functional interaction between miR-21 and p53 pathway in ischemic neuronal death is still rarely reported.

In the present study, the level of miR-21 decreased while p53 expression was elevated following ischemia. Overexpression of miR-21 protected against ischemic neuronal injury by inhibiting the p53/Bcl-2/Bax signaling pathway, while inhibition of miR-21 enhanced the p53/Bcl-2/Bax signaling and aggravated the ischemic neuronal injury. These novel findings first investigated the role of miR-21 in p53/Bcl-2/Bax pathway and characterized their interaction in ischemic neuronal injury, suggesting that miR-21/p53 may be attractive therapeutic molecules for treatment of ischemic stroke.

## RESULTS

### MiR-21 is down-regulated while p53 is up-regulated following ischemia *in vitro*

**Figure 1:**
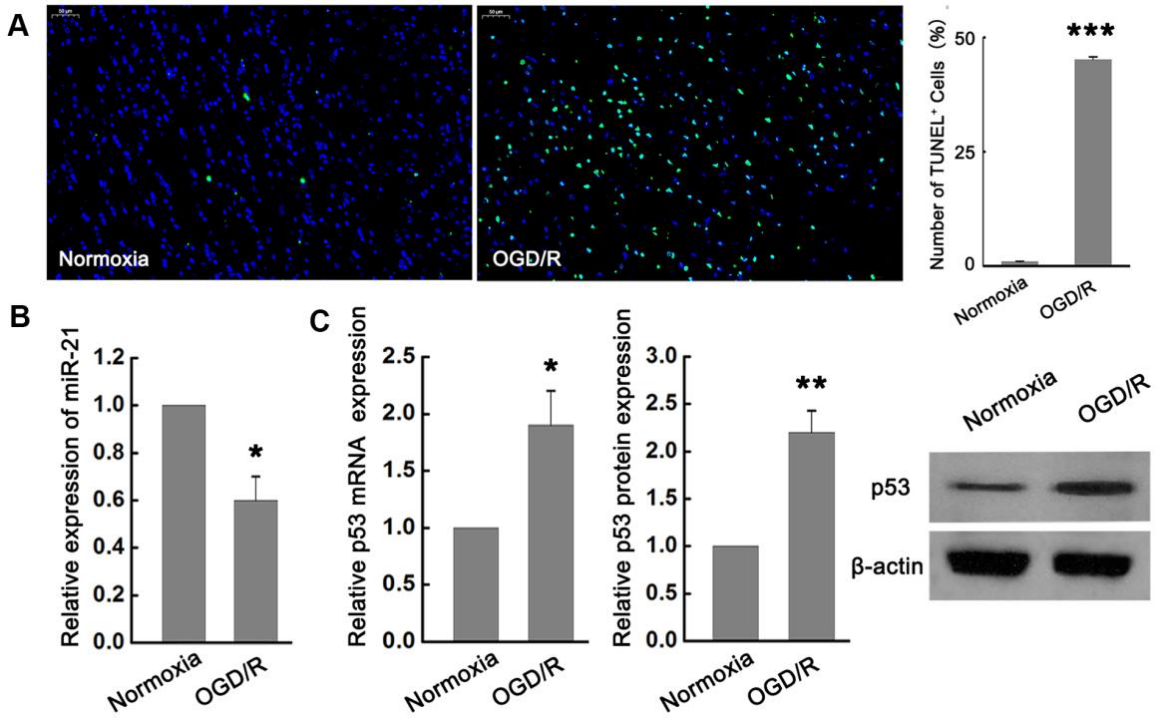
**miR-21 was down-regulated while p53 was up-regulated following ischemia *in vitro*.** (**A**) TUNEL assay showed that OGD/R-induced neuronal death significantly increased compared with the normoxia group. Scale bar, 50μm. (**B**) qRT-PCR revealed that miR-21 was remarkably down-regulated following OGD/R in neurons compared with the normoxia group. (**C**) qRT-PCR and western blot indicated that p53 mRNA and protein were both significantly up-regulated following OGD/R. All experiments were independently repeated three times. *, compared with normoxia group. *P≤0.05, **P≤0.01, ***P≤0.001.

In order to explore the potential role of miR-21 and p53 in ischemic neuronal injury, we examined the effect of OGD/R exposure on the expression of miR-21 and p53 in primary cultured mouse embryonic cortical neurons. TUNEL analysis showed that the neuronal death induced by OGD/R was significantly higher than that in normoxia group (Figure 1A), suggesting that OGD/R could induce apoptotic cell death *in vitro*. In the meantime, quantitative reverse transcription-polymerase chain reaction (qRT-PCR) revealed that miR-21 was remarkably down-regulated following OGD/R in neurons compared with the normoxia group (Figure 1B). In contrast to the expression change of miR-21 by OGD/R exposure, the expression of p53 mRNA and protein were both significantly up-regulated following OGD/R *in vitro* (Figure 1C).

### Overexpression of miR-21 down regulates p53 expression, while inhibition of miR-21 up regulates p53 expression following ischemia *in vitro*

**Figure 2:**
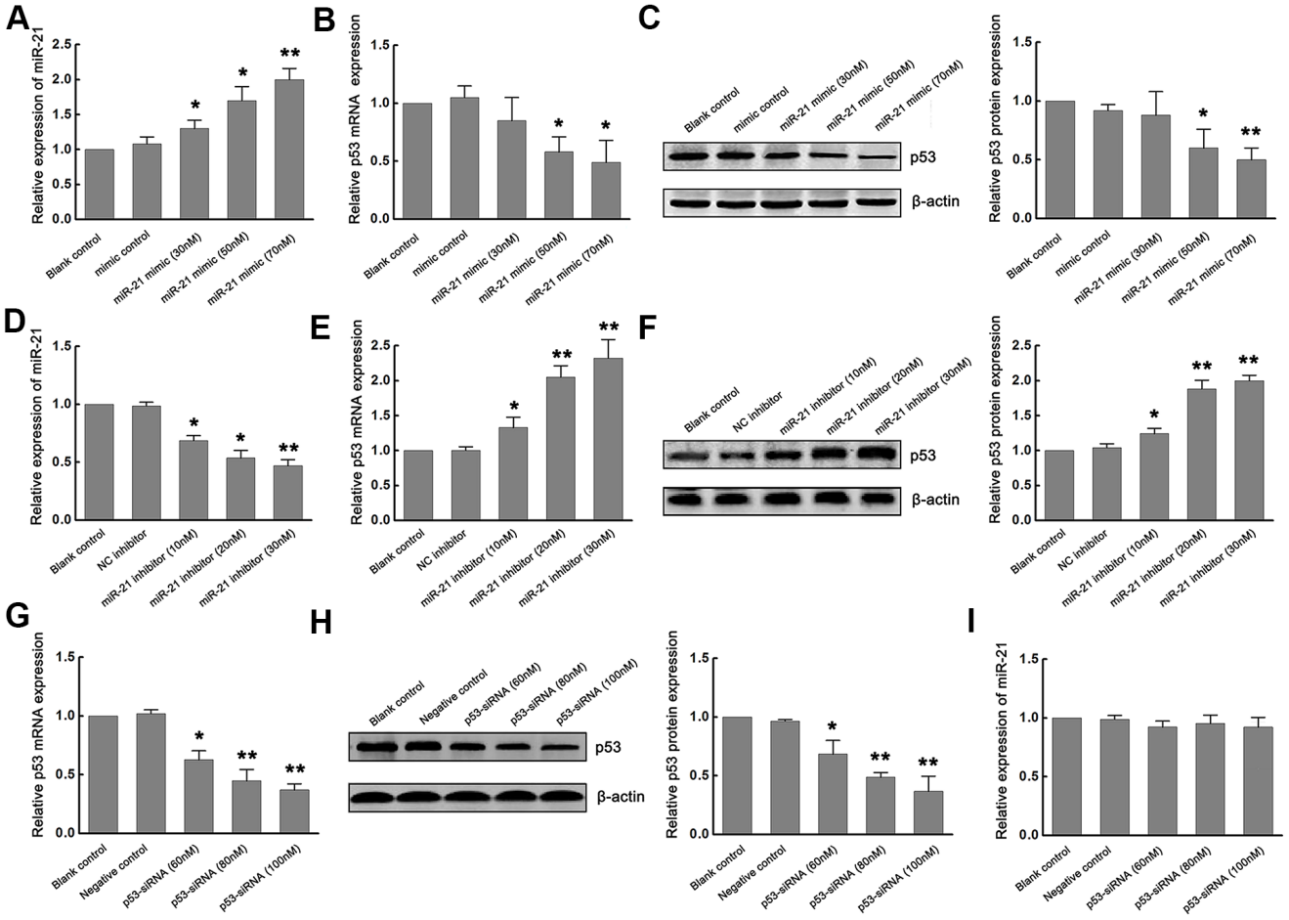
**Overexpression of miR-21 down regulated p53 expression, while inhibition of miR-21 up regulated p53 expression following ischemia *in vitro*.** (**A**) The modulation of miR-21 expression by transfecting different concentrations of miR-21 mimics into primary cultured neurons was confirmed by qRT-PCR. (**B**, **C**) The expression of p53 mRNA (**B**) and protein (**C**) in neurons decreased in a concentration-dependent manner with the increase of the concentration of miR-21 mimics. (**D**) The modulation of miR-21 expression by transfecting different concentrations of miR-21 inhibitors into primary cultured neurons was confirmed by qRT-PCR. (**E**, **F**) The expression of p53 mRNA (**E**) and protein (**F**) in neurons increased in a concentration-dependent manner with the increase of the concentration of miR-21 inhibitors. (**G**, **H**) The modulation of p53 mRNA (**G**) and protein (**H**) levels by transfecting different concentrations of p53-siRNAs into primary cultured neurons were confirmed by qRT-PCR and western blot. (**I**) qRT-PCR showed that the expression level of miR-21 did not change significantly with the decrease of p53 expression. All experiments were independently repeated three times. *, compared with blank control. *P≤0.05; **P≤0.01.

Since previous studies have reported that p53 mediated neuronal apoptosis, and miR-21 provided neuroprotection during ischemic injury [4, 8–11], we next determined whether miR-21 and p53 interact during ischemia *in vitro*. To elucidate the exact effect of miR-21 on the expression of p53 during ischemia *in vitro*, gain-of-function and loss-of-function experiments were carried out by transfecting different concentrations of miR-21 mimics (30nM, 50nM, 70nM) or miR-21 inhibitors (10nM, 20nM, 30nM) into primary cultured neurons and then treated with OGD/R. Neurons transfected with mimic control and treated with OGD/R were considered as the negative control of miR-21 mimic. Neurons transfected with negative control inhibitor (NC inhibitor) and treated with OGD/R were considered as the negative control of miR-21 inhibitor, while non-transfected neurons treated with OGD/R were identified as the blank control. Concentration-dependent up-regulation of miR-21 was detected in miR-21 mimic-transfected neurons, as confirmed by qRT-PCR (Figure 2A). In response to this, the expression of p53 mRNA and protein in neurons decreased in a concentration-dependent manner with the increase of the concentration of mR-21 mimic, as compared to the negative control and blank control (Figure 2B, 2C). Especially when the concentration of miR-21 mimic was 50 and 70 nM, the expression of p53 mRNA and protein was significantly down-regulated (Figure 2B, 2C), suggesting that overexpression of miR-21 inhibited p53 expression following ischemia *in vitro*. On the other hand, concentration-dependent down-regulation of miR-21 was detected in miR-21 inhibitor-transfected neurons, as confirmed by qRT-PCR (Figure 2D). Loss-of-function experiments showed that with the increase of the concentration of miR-21 inhibitor, the level of p53 increased in a concentration-dependent manner, including mRNA and protein levels (Figure 2E, 2F), suggesting that inhibition of miR-21 promoted p53 expression following ischemia *in vitro*.

We next assessed whether p53 regulated the expression of miR-21 during ischemia *in vitro*. Since p53 is up-regulated following OGD/R, loss-of-function experiments were carried out by transfecting different concentrations (60nM, 80nM, 100nM) of small interfering RNA (siRNA) targeting p53 (p53-siRNA) into primary cultured neurons and then treated with OGD/R. Neurons transfected with scramble RNA for p53-siRNA (p53-s-siRNA) and treated with OGD/R were considered as the negative control. Non-transfected neurons treated with OGD/R were identified as the blank control. qRT-PCR and western blot confirmed that p53 levels decreased in a concentration-dependent manner (Figure 2G, 2H). However, the expression level of miR-21 did not change significantly with the decrease of p53 expression (Figure 2I), suggesting that p53 may have no regulatory effect on miR-21 expression.

Based on the above results, overexpression of miR-21 down regulates p53 expression, while inhibition of miR-21 up regulates p53 expression following ischemia following ischemia *in vitro*, thus, p53 may be a downstream regulator of miR-21.

### MiR-21/p53 axis regulates Bcl-2/Bax following ischemia *in vitro*

**Figure 3:**
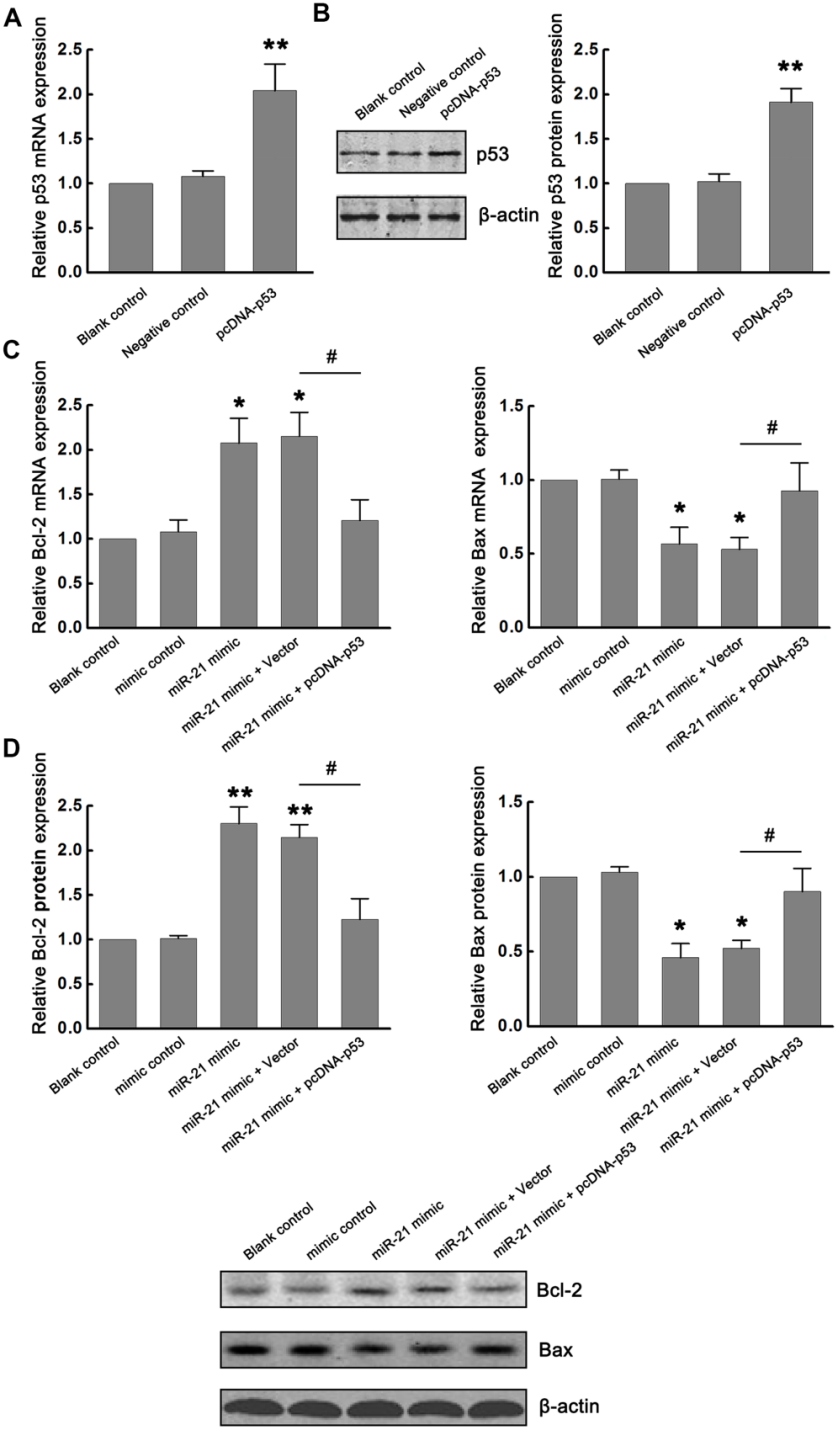
**miR-21/p53 axis regulated Bcl-2/Bax following ischemia *in vitro*.** (**A**, **B**) The modulation of p53 mRNA and protein expression by transfecting pcDNA-p53 into primary cultured neurons were confirmed by qRT-PCR (**A**) and western blot (**B**). (**C**, **D**) Effect of miR-21/p53 axis on the mRNA (**C**) and protein (**D**) expression of Bcl-2 and Bax. All experiments were independently repeated three times. *, compared with blank control. *P≤0.05, **P≤0.01. #, compared with miR-21 mimic + vector group. ^#^*P*≤0.05.

The p53 can promote apoptosis through interactions with Bcl-2 family proteins (such as Bax, a factor that promotes apoptosis, and Bcl-2, a factor that inhibits apoptosis). Based on the inhibitory effect of miR-21 on p53 expression, we further determined the functional interaction between miR-21 and p53/Bcl-2/Bax signaling following ischemia *in vitro*. Firstly, p53-expressing plasmids (pcDNA-p53) were constructed to up-regulate the expression of p53. Neurons transfected with vector (pcDNA) and treated with OGD/R were considered as the negative control. Non-transfected neurons treated with OGD/R were identified as the blank control. The modulation of p53 expression by transfecting pcDNA-p53 into primary cultured neurons was confirmed by qRT-PCR and western blot (Figure 3A, 3B). Then, we detected the effect of miR-21 on Bcl-2/Bax signaling. We found that overexpression of miR-21 in neurons inhibited the expression of Bax, while up-regulated the expression of Bcl-2 following OGD/R. To further validate the role of the miR-21/p53 axis in the regulation of Bcl-2/Bax, neurons were co-transfected with miR-21 mimic and pcDNA-p53 plasmid to detect the effect of p53 overexpression on the decrease of Bax and increase of Bcl-2 induced by miR-21 mimic following OGD/R. Notably, the introduction of pcDNA-p53 vector could weaken the inhibition of miR-21 mimic on Bax and the promotion of miR-21 mimic on Bcl-2 in neurons (Figure 3C, 3D). Taken together, the miR-21/p53 axis may regulate the expression of Bcl-2/Bax following ischemia *in vitro*.

### MiR-21 protects OGD/R-induced injury by inhibiting p53/Bcl-2/Bax signaling *in vitro*

**Figure 4:**
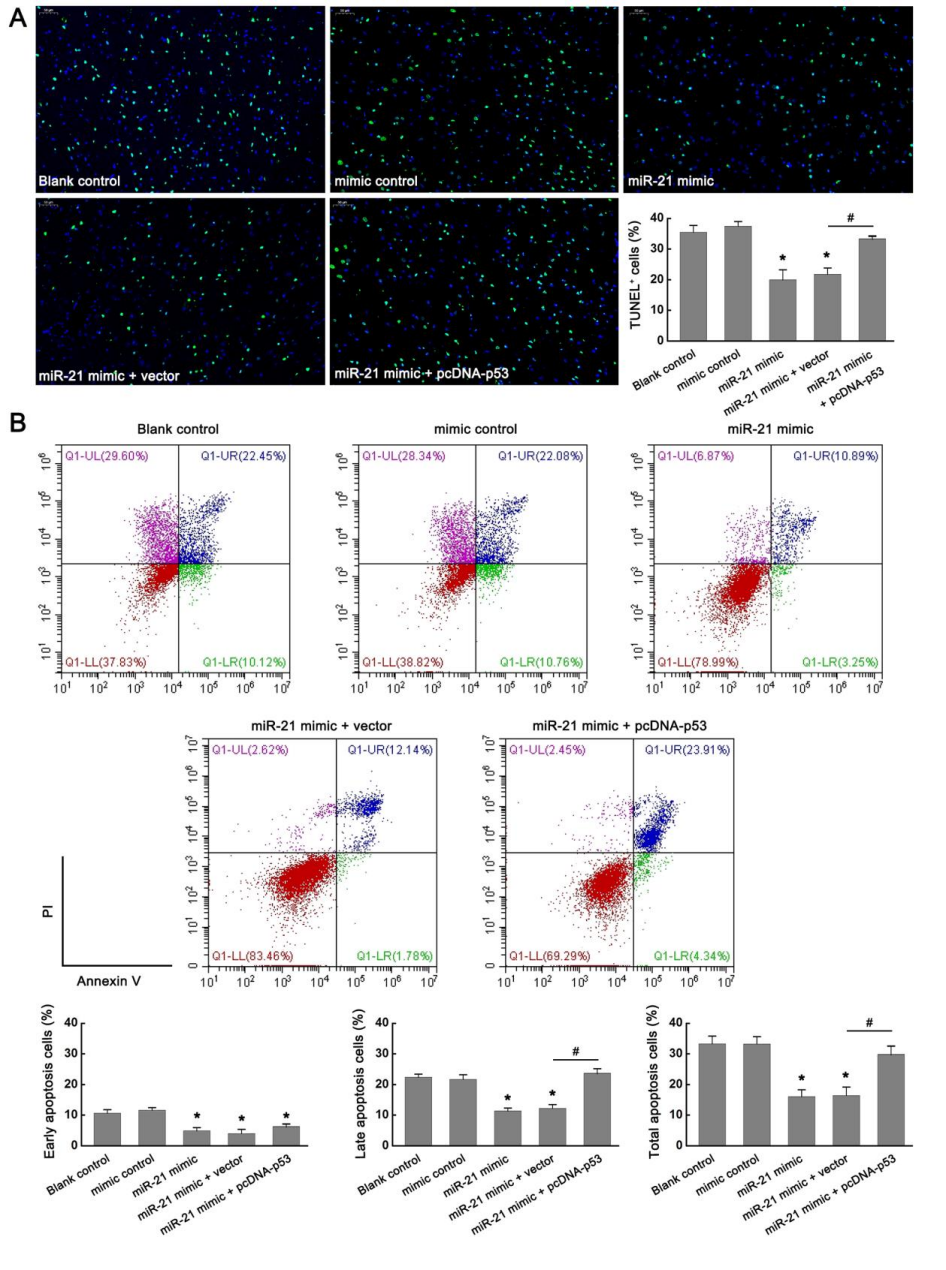
**miR-21 protected OGD/R-induced injury by inhibiting p53-Bcl-2/Bax signaling *in vitro*.** (**A**) Effect of miR-21/p53 axis on the percentage of TUNEL^+^ cells. Scale bar, 50μm. (**B**) Effect of miR-21/p53 axis on the percentage of early, late, and total apoptosis. All experiments were independently repeated three times. *, compared with blank control. *P≤0.05. #, compared with miR-21 mimic + vector group. ^#^*P*≤0.05.

To further validate the role of miR-21/p53 axis in the OGD/R-induced injury *in vitro*, TUNEL assay and flow cytometry based-Annexin V-FITC/PI double-staining were performed to test the cell apoptosis following OGD/R. From Figure 4A, TUNEL staining showed that up regulation of miR-21 significantly inhibited OGD/R-induced neuronal apoptosis, as reflected by the decreased percentage of TUNEL positive cells (TUNEL^+^) in the miR-21 mimic treatment group compared with that in the blank control group. However, the introduction of pcDNA-p53 (miR-21 mimic + pcDNA-p53 treatment group) reversed the inhibitory effect of miR-21 mimic on neuronal apoptosis (Figure 4A). Flow cytometry analysis was also performed to detect cell apoptosis, including the early and late apoptosis. Early apoptotic cells could be labeled by Annexin V-FITC staining (Figure 4B, the second quadrant), late apoptotic cells could be simultaneously labeled by Annexin V-FITC and propidium iodide (PI) (Figure 4B, the first quadrant), and living cells were neither labeled by Annexin V-FITC nor PI (Figure 4B, the third quadrant). Consistent with the results of TUNEL staining, flow cytometry analysis demonstrated that up-regulation of miR-21 in neuronal cells mitigated OGD/R-induced cell apoptosis compared with the blank control group (whether it is early apoptosis, late apoptosis rate, or total apoptosis), whereas the effect was attenuated by p53 overexpression (Figure 4B). Moreover, the introduction of pcDNA-p53 mainly reversed the inhibitory effect of miR-21 mimic on late apoptosis and total apoptosis (Figure 4B). Thus, the results demonstrated that overexpression of miR-21 protected OGD/R-induced cell apoptosis by targeting p53. Combined with the above results that miR-21/p53 axis regulated the expression of Bcl-2/Bax following OGD/R, we speculated that miR-21 may protect OGD/R-induced injury by inhibiting p53-Bcl-2/Bax signaling *in vitro*.

### MiR-21 regulates the p53-Bcl-2/Bax signaling following ischemia *in vivo*

Based on the above results, miR-21 may protect ischemic neuronal death by inhibiting the p53-Bcl-2/Bax signaling pathway *in vitro*. To further evaluate the role of miR-21/p53/Bcl-2/Bax pathway in ischemic neuronal damage following I/R *in vivo*, middle cerebral artery occlusion (MCAO) was performed to induce focal cerebral ischemia in mice. After modeling, the infarcted tissues in the ischemic core area were separated for qRT-PCR and western blot assays. The sham-operated animals were identified as the control group.

**Figure 5:**
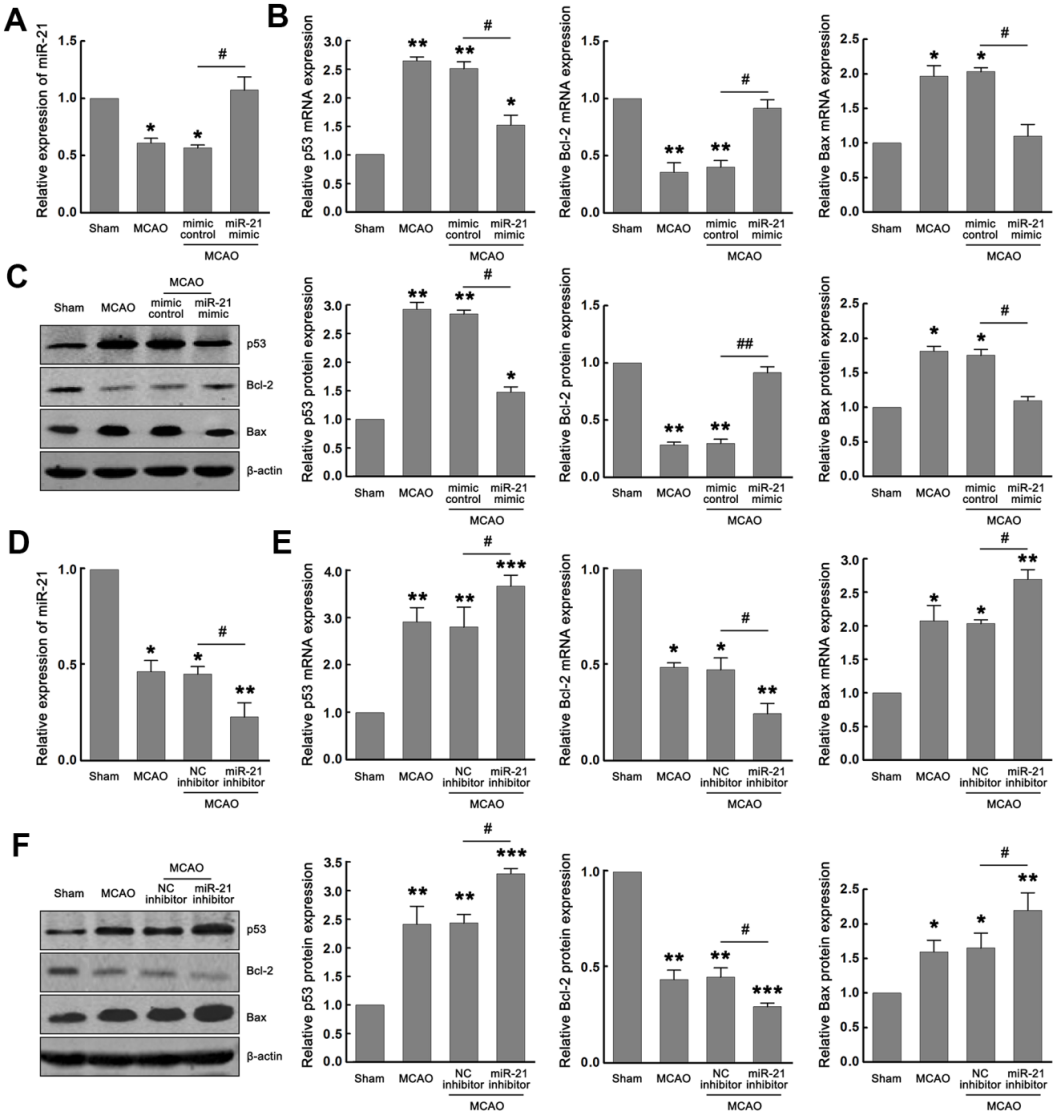
**MiR-21 regulated the p53/Bcl-2/Bax signaling following ischemia *in vivo*.** (**A**) qRT-PCR indicated the effect of miR-21 mimic and mimic control on the expression of miR-21 following I/R *in vivo*. (**B**, **C**) qRT-PCR (**B**) and western blot (**C**) indicated the effect of miR-21 mimic and mimic control on mRNA and protein levels of p53, Bax, and Bcl-2 following I/R *in vivo*. (**D**) qRT-PCR indicated the effect of miR-21 inhibitor and NC inhibitor on the expression of miR-21 following I/R *in vivo*. (**E**, **F**) qRT-PCR (**E**) and western blot (**F**) indicated the effect of miR-21 inhibitor and NC inhibitor on mRNA and protein levels of p53, Bax, and Bcl-2 following I/R *in vivo*. Eight mice were randomly selected from each treatment group. *, compared with the sham group. *P≤0.05, **P≤0.01, ***P≤0.001. #, mimic control + MCAO group vs. miR-21 mimic + MCAO group, or NC inhibitor + MCAO group vs. miR-21 inhibitor + MCAO group. ^#^*P*≤0.05, ^##^P≤0.01.

We first verified whether miR-21/p53/Bcl-2/Bax signaling pathway is activated following cerebral ischemia and whether miR-21 regulates the p53/Bcl-2/Bax signaling following ischemia *in vivo*. As expected, miR-21 and Bcl-2 were downregulated, while p53 and Bax were upregulated following I/R in the MCAO group compared with the sham group (Figure 5A–5F). To evaluate the effect of miR-21 expression on p53/Bcl-2/Bax signaling *in vivo*, miR-21 mimic, mimic control, miR-21 inhibitor, or NC inhibitor was injected into the lateral ventricle of mice before MCAO operation by using *in vivo* transfection reagent. After MCAO operation, the up-regulation of miR-21 in mice injected with miR-21 mimic compared with mimic control was confirmed by qRT-PCR (Figure 5A). Meanwhile, up regulation of miR-21 expression significantly reduced the mRNA and protein levels of p53 and Bax, and increased the mRNA and protein levels of Bcl-2 (Figure 5B–5C), which verified the regulatory effect of miR-21 mimic on p53/Bcl-2/Bax signaling pathway following I/R *in vivo*. The decline of miR-21 in mice injected with miR-21 inhibitor compared with NC inhibitor was also confirmed by qRT-PCR (Figure 5D). However, unlike the inhibitory effect of miR-21 mimic on p53/Bcl-2/Bax signaling, miR-21 inhibitor substantially promoted the expression of p53 and Bax, and down-regulated the expression of Bcl-2, including mRNA and protein levels (Figure 5E–5F). Thus, both the gain-of-function experiment and the loss-of-function experiment suggested that miR-21 regulated the p53/Bcl-2/Bax signaling following ischemia *in vivo*.

### MiR-21/p53/Bcl-2/Bax signaling regulates ischemic neuronal injury *in vivo*

**Figure 6:**
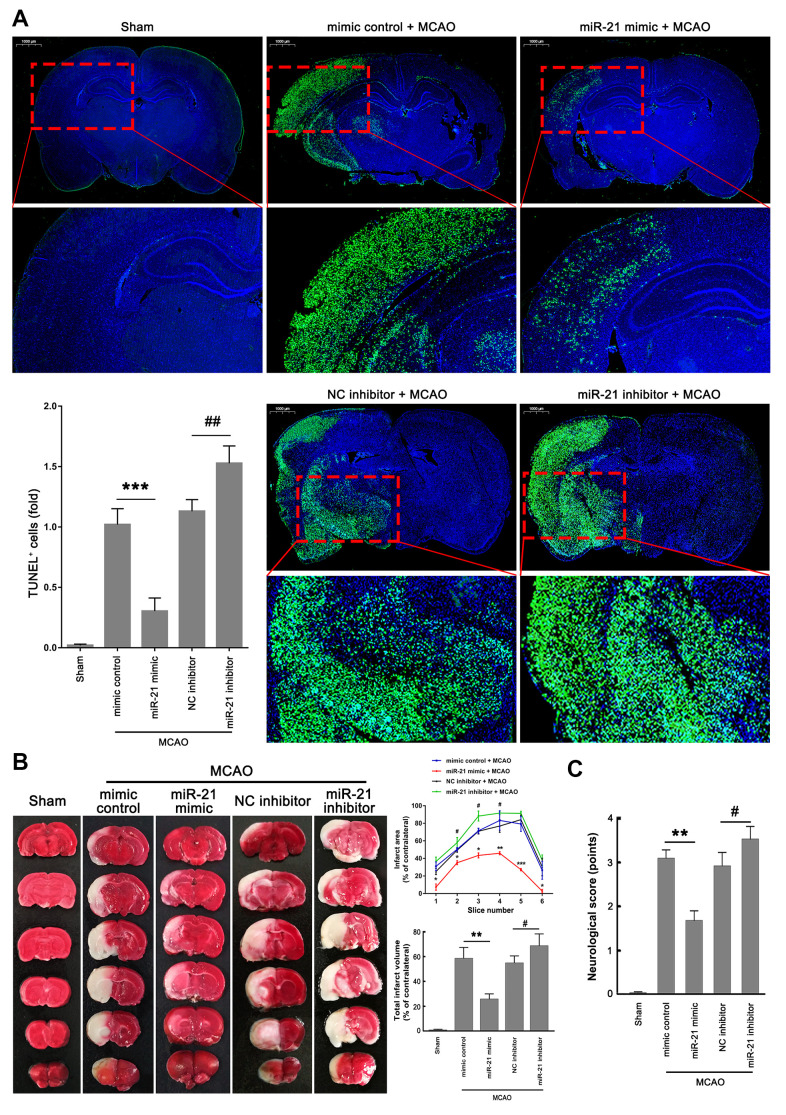
**MiR-21/p53/Bcl-2/Bax signaling regulates ischemic neuronal injury *in vivo*.** (**A**) Representative images showed the TUNEL-labeled cells in the brain slices. Bar graph summarized the numbers of TUNEL^+^ cells (fold) in the ischemic region. Scale bar, 1000μm. (**B**) Brain infarction was visualized by TTC staining at 24 h after operation. Curve lines summarized infarct areas in the ipsilateral hemisphere normalized to the total areas of the contralateral hemisphere in sequential coronal brain slices. Bar graph showed the volumes of total cerebral infarct in the ipsilateral hemisphere normalized to the total volumes of the contralateral hemisphere. (**C**) Neurologic deficits were analyzed by a neurologic deficit score. Eight mice were randomly selected from each treatment group. *, mimic control + MCAO group vs. miR-21 mimic + MCAO group. *P≤0.05, **P≤0.01, ***P≤0.001. #, NC inhibitor + MCAO group vs. miR-21 inhibitor + MCAO group. ^#^*P*≤0.05, ^##^*P*≤0.01.

Given that miR-21 regulated the p53/Bcl-2/Bax signaling following ischemia *in vivo*, we next further extended our analysis of the miR-21/p53/Bcl-2/Bax signaling pathway in ischemic damage and neurological functions. Much fewer TUNEL^+^ cells were detected in the ischemic regions in mice injected with miR-21 mimic compared with mimic control treatment group (Figure 6A), but a remarkable increase of TUNEL^+^ cells was observed in the ischemic regions in mice injected with miR-21 inhibitor compared with NC inhibitor treatment group (Figure 6A), suggesting that overexpression of miR-21 *in vivo* effectively reduced ischemic neuronal death, while inhibition of miR-21 *in vivo* further aggravates the ischemic neuronal death following I/R. 2,3,5-Triphenyltetrazolium chloride (TTC) staining was performed to visualize the ischemic lesions (unstained brain areas). It is worth noting that injection of miR-21 mimic significantly improved the volume of cerebral infarction induced by I/R, as reflected by the reduction of infarct areas in each coronal brain slice or the total cerebral infarct volumes, thus protecting the brain from ischemic injury (Figure 6B). However, inhibition of miR-21 notably increased I/R-induced infarct volume, resulting in the aggravation of ischemic neuronal injury (Figure 6B). In addition, the neurological scoring system was used to assess the neurological deficits following I/R at 24 h reperfusion. As shown in Figure 6C, mice injected with miR-21 mimic revealed a better neurological status following ischemia compared with the mimic control treatment group, as reflected by the decreased neurologic deficit score in the miR-21 mimic treatment group. Nevertheless, mice injected with miR-21 inhibitor showed a worse neurological status with a higher neurologic deficit score compared with the NC inhibitor treatment group. Taken together, overexpression of miR-21 protected ischemic neuronal injury and improved neurological deficits *in vivo*, while inhibition of miR-21 further aggravated the ischemic neuronal injury. Combined with the effect of miR-21 expression on p53/Bcl-2/Bax signaling following I/R, we speculated that miR-21/p53/Bcl-2/Bax signaling may regulate ischemic neuronal injury *in vivo*.

## DISCUSSION

Various miRNAs have been demonstrated to regulate important target genes involved in stroke [18, 19]. Among the reported miRNAs, miR-21 is a key factor in the pathogenesis of different types of strokes [19]. For ischemic stroke, some previous studies have shown that miR-21, as an important miRNA promoting survival and inhibiting apoptosis in the development of cerebral ischemia, might be a potential diagnostic biomarker or therapeutic target for this type of stroke [4, 8, 9, 20]. In this study, miR-21 was down-regulated following ischemia *in vitro* and *in vivo*, overexpression of miR-21 alleviated OGD/R or MCAO/R-induced neuronal injury, while inhibition of miR-21 aggravated the ischemic neuronal injury, further demonstrating the protective effect of miR-21 on ischemic stroke. However, we found a problem worthy of discussion. Buller et al. found that miR-21 was significantly elevated in the ischemic boundary zone [9], here, our results indicated a decreased expression of miR-21 in the tissues isolated from ischemic core, which was opposite to that in the ischemic boundary zone. Since the ischemic hemisphere consists of ischemic core, ischemic boundary zone, and non-ischemic areas, expression patterns for some molecules may vary in different brain areas. Previous studies have observed significantly different or diametrically opposite patterns of gene expression between the ischemic core and penumbra [21, 22]. Thus, the expression pattern for miR-21 may vary in different brain regions following ischemia, and the differential expression of miR-21 between ischemic core and penumbra may have guiding significance for the selection of ischemic intervention strategies.

p53 is a transcription factor that is widely known as a tumor suppressor protein. In recent years, the p53 pathway has been demonstrated to be one of the mechanisms of regulating apoptosis in response to multiple signals [23]. Mechanically, p53-mediated apoptosis can be triggered by multiple miRNAs [23]. Several studies have reported the simultaneous occurrence of both miR-21 overexpression and p53 functional impairment in some cancer types, and miR-21 could inhibit the activity or expression level of many genes in p53 signaling network [24–26], which suggested a possible interaction between miR-21 and p53. Moscetti et al. evidenced the direct interaction between miR-21-3p and p53 DNA Binding Domain (p53-DBD) *in vitro*, providing a possible role of miR-21-3p in p53 functional impairment [27]. Although studies have reported the impact of miR-21 on p53 and multiple genes in the p53 network, most of the research focused on the role of miR-21/p53 signaling in various tumors [24–26]. However, the crosstalk between p53 and miR-21 in ischemic stroke remains unclear. In the present study, p53 was up-regulated following ischemia *in vitro* and *in vivo*, which was contrary to that of miR-21. And p53 was a downstream regulator of miR-21, overexpression of miR-21 inhibited the expression of p53 following ischemia *in vitro* and *in vivo,* while inhibition of miR-21 *in vitro* and *in vivo* promoted p53 expression following ischemia, suggesting miR-21/p53 axis may play a potential role in ischemia.

The main way of p53 mediated apoptosis is to promote the gene transcription of downstream pro-apoptotic factors, which in turn triggers mitochondrial pathways [23]. Mitochondrial dysfunction mediated by Bcl-2 family is an important event of apoptosis [28]. Bcl-2, which inhibits apoptosis, and Bax, which promotes apoptosis, are representatives of the Bcl-2 family. p53/Bcl-2/Bax interaction has been approved to trigger cell apoptosis in multiple diseases [28, 29]. However, the functional interaction between miR-21 and p53/Bcl-2/Bax signaling following ischemia has not been explored. Here, our results indicated that overexpression of miR-21 in neuronal cells inhibited Bax expression, while promoted Bcl-2 expression following OGD/R and I/R. Moreover, the effect of miR-21 up-regulation on Bax and Bcl-2 expression could be attenuated by overexpression of p53 at the same time, further confirming that the miR-21/p53 axis may regulate the expression of Bcl-2/Bax following ischemia *in vitro*. Based on the above results, we speculated that miR-21/p53/Bcl-2/Bax signaling may be involved in OGD/R-induced neuronal injury. As we expected, up-regulation of miR-21 significantly mitigated OGD/R-induced neuronal apoptosis, whereas the effect was attenuated by p53 overexpression at the same time, providing the evidence that miR-21 protected OGD/R-induced injury by inhibiting p53/Bcl-2/Bax signaling. Accordingly, the above results seems to point to the importance of miR-21 in the protection of ischemic injury by targeting p53/Bcl-2/Bax signaling.

To extend this analysis to neuroprotection and cerebral ischemia treatment, overexpression or inhibition of miR-21 *in vivo* was performed to detect the effect of miR-21 on p53/Bcl-2/Bax signaling and cerebral ischemic damage. The results indicated that up regulation of miR-21 significantly decreased the level of Bax and increased the level of Bcl-2 *in vivo*. Meanwhile, ischemic neuronal death was also abolished by miR-21 up-regulation. It is worth noting that overexpression of miR-21 significantly reduced I/R-induced brain infarct volume and improved neurological deficits following I/R, suggesting the regulatory effect of miR-21/p53/Bcl-2/Bax signaling in ischemic injury. Moreover, inhibiting the expression of miR-21 substantially promoted the expression of p53 and Bax, down-regulated the expression of Bcl-2, and aggravated the ischemic neuronal injury *in vivo*, which further verified the regulatory effect of miR-21/p53/Bcl-2/Bax signaling in ischemic injury.

In conclusion, this study represents the first time that miR-21 regulates ischemic neuronal injury via the p53/Bcl-2/Bax signaling pathway. Thus, we uncover a novel mechanism of miR-21 in regulating cerebral ischemic injury, suggesting that miR-21/p53 may be attractive therapeutic molecules for treatment of ischemic stroke. However, this study also has some limitations. Firstly, it was reported that there was no miR-21 binding site in the p53 3’UTR in either human or mouse [25], yet Moscetti et al. evidenced the direct interaction between miR-21-3p and p53-DBD [27]. So whether miR-21 represses the expression of p53 following ischemia by binding with p53-DBD still needs to be verified by more molecular biological experiments. Secondly, although we indicated that miR-21 protected against ischemic neuronal injury by inhibiting the p53/Bcl-2/Bax signaling pathway, we didn’t exclude the existence of other miR-21 or p53 targets that may be involved in ischemic neuronal damage.

## MATERIALS AND METHODS

### Animals

Adult male C57BL/6 mice at about 90 days old of age were used for *in vivo* study. The mice were housed under standard housing conditions. All animal experiments were approved by the Institutional Animal Care and Use Committee of Renmin Hospital of Wuhan University.

### Cell culture

The cerebral cortex was isolated from E16-18 mice embryos on ice. Primary cortical neurons were enzymatically digested by using trypsin (Invitrogen, Shanghai, China). Then the dissociated cells were seeded on coverslips or plates coated with poly D-lysine (PDL). Cells were first cultured in DMEM/F12 medium (Invitrogen) containing 10% FBS. After 4h of culture, half of the medium was replaced by Neurobasal medium (Invitrogen) supplemented with 4% B27. After that, replaced half of the medium with fresh medium every 3 days.

### OGD/R

For OGD treatment, cortical neurons (after culture for 10-12 days) were cultured in glucose-free bicarbonate buffer (pH 7.4) and placed in a hypoxia chamber for 3 h. After challenged with OGD, the glucose-free bicarbonate buffer was removed. Neurons were grown in fresh complete culture medium and incubated in 5% CO_2_ at 37° C for 24 h to achieve reoxygenation. Cells in the normoxic group were not subjected to OGD.

### TUNEL assay

The cultured neurons were fixed with 4% formaldehyde. Brain tissues were fixed, paraffin embedded and cut at 4 μm. Primary cortical neurons or brain slices were placed in a dark box and incubated with TUNEL staining solution at room temperature for 1 h. The number of TUNEL-positive cells was counted with fluorescence microscopy and quantified by using Image J software.

### RNA extraction and qRT-PCR

Total RNAs were extracted by using TRIzol reagent (Invitrogen). After the RNAs were reversely transcribed to cDNA, the mRNA level of p53, Bcl-2 and Bax were examined by qRT-PCR with SYBR Green Real-Time PCR Master Mixes (ThermoFisher, Waltham, MA, USA). For miR-21 analysis, TaqMan miRNA assay kit (ThermoFisher) was used to quantify miRNA expression. U6 and GAPDH were used as the internal control to normalize gene expression.

### Western blot assay

The total protein was extracted with RIPA lysis buffer. Western blot assay was performed as described previously [4, 14]. The antibodies used in this paper are as follows: anti-p53 antibody (catalogue number sc-126, 1: 500 dilution; Santa Cruz, CA, USA), anti-Bax antibody (catalogue number sc-7480, 1: 500 dilution; Santa Cruz), anti-Bcl-2 antibody (catalogue number sc-7382, 1: 500 dilution; Santa Cruz), and anti-β-actin (catalogue number sc-8432, 1: 1000 dilution; Santa Cruz). LI-COR Odyssey Imaging System was applied to image and analyze the protein bands. The protein levels of p53, Bax and Bcl-2 were normalized to that of β-actin.

### Cell transfection

miR-21 mimic was designed to overexpressed miR-21 expression, and mimic control was considered as the negative control of miR-21 mimic. miR-21 inhibitor was designed to inhibit miR-21 expression, and negative control inhibitor (NC inhibitor) was considered as the negative control of miR-21 inhibitor. p53-siRNA has been designed to specifically silence p53 expression. p53-s-siRNA was considered as the negative control of p53-siRNA. The p53 expressing plasmid (pcDNA-p53) was constructed by cloning the p53 sequence into a pcDNA vector. The pcDNA empty vector was considered as the negative control of pcDNA-p53. Cortical neurons were transfected with above reagents by using Lipofectamine 2000 (Invitrogen).

### Flow cytometry analysis

Apoptosis was also detected by flow cytometry analysis. The neuronal cells were stained with FITC-conjugated Annexin V and PI in no-light condition by using the Annexin V-FITC/PI Apoptosis Detection Kit (Vazyme Biotech, Tianjin, China). After 15 min of staining, the cells were analyzed by using a Beckman Coulter FC500 flow cytometer.

### Stereotaxic injection and MCAO model

Firstly, stereotaxic injection of miR-21 mimic or mimic control into the lateral ventricle of mice was performed. After anesthesia, mice were fixed on a stereotaxic apparatus. 5 μl of miR-21 mimic (or mimic control) and 5 μl of *in vivo* transfection reagent (Entranster™-*in vivo*; Engreen, Beijing, China) were mixed and injected into the lateral ventricle of mice. One day post-injection, MCAO was performed as described previously [4, 14]. After 60 min of occlusion, the MCAO suture was removed, followed by reperfusion for 24h. The sham-operated mice received the same operations as the MCAO group except without stereotaxic injection and the MCAO suture insertion.

### TTC staining

TTC staining was applied to measure the volume of cerebral infarction. The mice were sacrificed and quickly removed the brains at 24 h post MCAO or sham operations. Then, freeze the brain at -20° C for 30 min quickly. The frozen brain was sliced into 6 slices along the coronal plane on ice. Afterward, coronal sections were placed in 1% TTC solution, coverd with tin foil, then incubated at 37° C for 30 minutes, and fixed overnigh. Infarct volume was calculated by Image J software.

### Neurologic deficit score

A neurologic deficit score was performed to assess the neurological status of mice following ischemia at 24 h reperfusion. 0: no neurological deficits; 1: the front paws on the paralyzed side can not be fully extended; 2: turning to the paralyzed side when walking; 3: dumping to the paralyzed side when walking; 4: impaired consciousness or unable to spontaneously walk.

### Statistical analysis

All data are expressed as mean ± standard deviation (SD) of 3 independent experiment. The main parameter tests were *t*-tests and one-way ANOVAs. *P*<0.05 was considered statistically significant.

## References

[1] Manzanero S, Santro T, Arumugam TV. Neuronal oxidative stress in acute ischemic stroke: sources and contribution to cell injury. Neurochem Int. 2013; 62:712–18. 10.1016/j.neuint.2012.11.009 23201332

[2] Zhang A, Qian Y, Qian J. MicroRNA-152-3p protects neurons from oxygen-glucose-deprivation/reoxygenation-induced injury through upregulation of Nrf2/ARE antioxidant signaling by targeting PSD-93. Biochem Biophys Res Commun. 2019; 517:69–76. 10.1016/j.bbrc.2019.07.012 31326116

[3] Lai TW, Zhang S, Wang YT. Excitotoxicity and stroke: identifying novel targets for neuroprotection. Prog Neurobiol. 2014; 115:157–88. 10.1016/j.pneurobio.2013.11.006 24361499

[4] Yan H, Rao J, Yuan J, Gao L, Huang W, Zhao L, Ren J. Long non-coding RNA MEG3 functions as a competing endogenous RNA to regulate ischemic neuronal death by targeting miR-21/PDCD4 signaling pathway. Cell Death Dis. 2017; 8:3211. 10.1038/s41419-017-0047-y 29238035 PMC5870589

[5] Pillai RS. MicroRNA function: multiple mechanisms for a tiny RNA? RNA. 2005; 11:1753–61. 10.1261/rna.2248605 16314451 PMC1370863

[6] Sun Y, Gui H, Li Q, Luo ZM, Zheng MJ, Duan JL, Liu X. MicroRNA-124 protects neurons against apoptosis in cerebral ischemic stroke. CNS Neurosci Ther. 2013; 19:813–19. 10.1111/cns.12142 23826665 PMC6493643

[7] Liu W, Chen X, Zhang Y. Effects of microRNA-21 and microRNA-24 inhibitors on neuronal apoptosis in ischemic stroke. Am J Transl Res. 2016; 8:3179–87. 27508039 PMC4969455

[8] Zhou J, Zhang J. Identification of miRNA-21 and miRNA-24 in plasma as potential early stage markers of acute cerebral infarction. Mol Med Rep. 2014; 10:971–76. 10.3892/mmr.2014.2245 24841240

[9] Buller B, Liu X, Wang X, Zhang RL, Zhang L, Hozeska-Solgot A, Chopp M, Zhang ZG. MicroRNA-21 protects neurons from ischemic death. FEBS J. 2010; 277:4299–307. 10.1111/j.1742-4658.2010.07818.x 20840605 PMC2957309

[10] Zhang T, Wang H, Li Q, Fu J, Huang J, Zhao Y. MALAT1 Activates the P53 Signaling Pathway by Regulating MDM2 to Promote Ischemic Stroke. Cell Physiol Biochem. 2018; 50:2216–28. 10.1159/000495083 30419554

[11] Hong LZ, Zhao XY, Zhang HL. p53-mediated neuronal cell death in ischemic brain injury. Neurosci Bull. 2010; 26:232–40. 10.1007/s12264-010-1111-0 20502500 PMC5560294

[12] Wu Z, Wu P, Zuo X, Yu N, Qin Y, Xu Q, He S, Cen B, Liao W, Ji A. LncRNA-N1LR Enhances Neuroprotection Against Ischemic Stroke Probably by Inhibiting p53 Phosphorylation. Mol Neurobiol. 2017; 54:7670–85. 10.1007/s12035-016-0246-z 27844279

[13] Zhang L, Wang H. Long Non-coding RNA in CNS Injuries: A New Target for Therapeutic Intervention. Mol Ther Nucleic Acids. 2019; 17:754–66. 10.1016/j.omtn.2019.07.013 31437654 PMC6709344

[14] Yan H, Yuan J, Gao L, Rao J, Hu J. Long noncoding RNA MEG3 activation of p53 mediates ischemic neuronal death in stroke. Neuroscience. 2016; 337:191–99. 10.1016/j.neuroscience.2016.09.017 27651151

[15] He L, He X, Lim LP, de Stanchina E, Xuan Z, Liang Y, Xue W, Zender L, Magnus J, Ridzon D, Jackson AL, Linsley PS, Chen C, et al. A microRNA component of the p53 tumour suppressor network. Nature. 2007; 447:1130–34. 10.1038/nature05939 17554337 PMC4590999

[16] Afanasyeva EA, Mestdagh P, Kumps C, Vandesompele J, Ehemann V, Theissen J, Fischer M, Zapatka M, Brors B, Savelyeva L, Sagulenko V, Speleman F, Schwab M, Westermann F. MicroRNA miR-885-5p targets CDK2 and MCM5, activates p53 and inhibits proliferation and survival. Cell Death Differ. 2011; 18:974–84. 10.1038/cdd.2010.164 21233845 PMC3131937

[17] Jin L, Hu WL, Jiang CC, Wang JX, Han CC, Chu P, Zhang LJ, Thorne RF, Wilmott J, Scolyer RA, Hersey P, Zhang XD, Wu M. MicroRNA-149*, a p53-responsive microRNA, functions as an oncogenic regulator in human melanoma. Proc Natl Acad Sci USA. 2011; 108:15840–45. 10.1073/pnas.1019312108 21896753 PMC3179083

[18] Mirzaei H, Momeni F, Saadatpour L, Sahebkar A, Goodarzi M, Masoudifar A, Kouhpayeh S, Salehi H, Mirzaei HR, Jaafari MR. MicroRNA: Relevance to stroke diagnosis, prognosis, and therapy. J Cell Physiol. 2018; 233:856–65. 10.1002/jcp.25787 28067403

[19] Panagal M, Biruntha M, Vidhyavathi RM, Sivagurunathan P, Senthilkumar SR, Sekar D. Dissecting the role of miR-21 in different types of stroke. Gene. 2019; 681:69–72. 10.1016/j.gene.2018.09.048 30267810

[20] Xiang Y, Guo J, Peng YF, Tan T, Huang HT, Luo HC, Wei YS. Association of miR-21, miR-126 and miR-605 gene polymorphisms with ischemic stroke risk. Oncotarget. 2017; 8:95755–63. 10.18632/oncotarget.21316 29221163 PMC5707057

[21] Hori M, Nakamachi T, Shibato J, Rakwal R, Tsuchida M, Shioda S, Numazawa S. PACAP38 differentially effects genes and CRMP2 protein expression in ischemic core and penumbra regions of permanent middle cerebral artery occlusion model mice brain. Int J Mol Sci. 2014; 15:17014–34. 10.3390/ijms150917014 25257527 PMC4200817

[22] Xu Q, Deng F, Xing Z, Wu Z, Cen B, Xu S, Zhao Z, Nepomuceno R, Bhuiyan MI, Sun D, Wang QJ, Ji A. Long non-coding RNA C2dat1 regulates CaMKIIδ expression to promote neuronal survival through the NF-κB signaling pathway following cerebral ischemia. Cell Death Dis. 2016; 7:e2173. 10.1038/cddis.2016.57 27031970 PMC4823958

[23] Pei L, Shang Y, Jin H, Wang S, Wei N, Yan H, Wu Y, Yao C, Wang X, Zhu LQ, Lu Y. DAPK1-p53 interaction converges necrotic and apoptotic pathways of ischemic neuronal death. J Neurosci. 2014; 34:6546–56. 10.1523/JNEUROSCI.5119-13.2014 24806680 PMC6608141

[24] Liu Z, Lu Y, Xiao Y, Lu Y. Upregulation of miR-21 expression is a valuable predicator of advanced clinicopathological features and poor prognosis in patients with renal cell carcinoma through the p53/p21-cyclin E2-Bax/caspase-3 signaling pathway. Oncol Rep. 2017; 37:1437–44. 10.3892/or.2017.5402 28184919

[25] Ma X, Choudhury SN, Hua X, Dai Z, Li Y. Interaction of the oncogenic miR-21 microRNA and the p53 tumor suppressor pathway. Carcinogenesis. 2013; 34:1216–23. 10.1093/carcin/bgt044 23385064 PMC3670255

[26] Wei X, You X, Zhang J, Zhou C. miR-21 inhibitor facilitates the anticancer activity of doxorubicin loaded nanometer in melanoma. Oncol Rep. 2019; 42:414–24. 10.3892/or.2019.7167 31115580

[27] Moscetti I, Cannistraro S, Bizzarri AR. Probing direct interaction of oncomiR-21-3p with the tumor suppressor p53 by fluorescence, FRET and atomic force spectroscopy. Arch Biochem Biophys. 2019; 671:35–41. 10.1016/j.abb.2019.05.026 31181181

[28] Beberok A, Wrześniok D, Rok J, Rzepka Z, Respondek M, Buszman E. Ciprofloxacin triggers the apoptosis of human triple-negative breast cancer MDA-MB-231 cells via the p53/Bax/Bcl-2 signaling pathway. Int J Oncol. 2018; 52:1727–37. 10.3892/ijo.2018.4310 29532860

[29] Duan P, Hu C, Butler HJ, Quan C, Chen W, Huang W, Tang S, Zhou W, Yuan M, Shi Y, Martin FL, Yang K. 4-Nonylphenol induces disruption of spermatogenesis associated with oxidative stress-related apoptosis by targeting p53-Bcl-2/Bax-Fas/FasL signaling. Environ Toxicol. 2017; 32:739–53. 10.1002/tox.22274 27087316

